# Complete genomes of Hairstreak butterflies, their speciation, and nucleo-mitochondrial incongruence

**DOI:** 10.1038/srep24863

**Published:** 2016-04-28

**Authors:** Qian Cong, Jinhui Shen, Dominika Borek, Robert K. Robbins, Zbyszek Otwinowski, Nick V. Grishin

**Affiliations:** 1 Department of Biophysics and Biochemistry, University of Texas Southwestern Medical Center, 5323 Harry Hines Boulevard, Dallas, Texas 75390-8816, USA; 2 Department of Entomology, National Museum of Natural History, PO Box 37012, NHB Stop 105, Smithsonian Institution, Washington, D.C., 20013-7012 USA; 3 Howard Hughes Medical Institute, University of Texas Southwestern Medical Center, 5323 Harry Hines Boulevard, Dallas, Texas 75390-9050, USA

## Abstract

Comparison of complete genomes of closely related species enables research on speciation and how phenotype is determined by genotype. Lepidoptera, an insect order of 150,000 species with diverse phenotypes, is well-suited for such comparative genomics studies if new genomes, which cover additional Lepidoptera families are acquired. We report a 729 Mbp genome assembly of the *Calycopis cecrops*, the first genome from the family Lycaenidae and the largest available Lepidoptera genome. As detritivore, *Calycopis* shows expansion in detoxification and digestion enzymes. We further obtained complete genomes of 8 *Calycopis* specimens: 3 *C. cecrops* and 5 *C. isobeon*, including a dry specimen stored in the museum for 30 years. The two species differ subtly in phenotype and cannot be differentiated by mitochondrial DNA. However, nuclear genomes revealed a deep split between them. Genes that can clearly separate the two species (speciation hotspots) mostly pertain to circadian clock, mating behavior, transcription regulation, development and cytoskeleton. The speciation hotspots and their function significantly overlap with those we previously found in *Pterourus*, suggesting common speciation mechanisms in these butterflies.

Recent advances in sequencing techniques open unprecedented opportunities for addressing biological questions by comparative genomics. The relative ease of obtaining complete Eukaryotic genomes for a group of organisms enables researchers to approach questions about speciation, phylogeny and genotypic determinants of phenotypic traits. Butterflies are particularly suited for such analyses due to their phenotypic diversity, relative genotypic simplicity, extensive knowledge of their biology, and phylogenetic closeness to the model organism *Drosophila*. Among butterflies, representative genomes are currently known for four families: the swallowtails (Papilionidae)^1^,^2^,^3^, the brushfoots (Nymphalidae)^4^,^5^,^6^, the whites and sulphurs (Pieridae)^7^ and the skippers (Hesperiidae)^8^. The brushfoots have been prevalent in genomics studies, with research on *Heliconius* and the Monarch (*Danaus plexippus*) leading the field^9^,^10^. For comparative genomics of butterflies, it is essential to sequence complete genomes of all major phylogenetic groups.

Lycaenidae is the second-largest family of butterflies and hairstreaks (Theclinae) form its major subfamily. To break the ground of Lycaenidae genomics, we chose the genus *Calycopis*. While species-rich in the Neotropics, *Calycopis* in the United States is traditionally divided into two species: Red-Banded Groundstreak *C. cecrops* and Dusky-Blue Groundstreak *C. isobeon*. *C. cecrops* is a common species in the eastern half of the U. S. from Michigan and New York to Florida. In Texas and neighboring states, *C. cecrops* overlaps in distribution with *C. isobeon*, which ranges southwards into Mexico to Panama^11^.

*C. cecrops* and *C. isobeon* were traditionally considered to be close relatives. They are difficult to tell apart, and no identification feature is absolute, especially in their region of sympatry. They mainly differ in the wing patterns beneath (Fig. 1), although individuals with intermediate wing patterns occur where the two species meet in east-central Texas^11^,^12^. Compared to *C. isobeon*, *C. cecrops* is characterized by broader red bands on the wings (especially on the forewing), and larger black spots with smaller red caps near the tornus of the hindwing. While most butterfly species can be identified by inspection of male genitalia, genitalia of *C. cecrops* and *C. isobeon* are very similar, although penis length varies clinally, getting shorter southwards^11^. Moreover, their COI mitochondrial barcode sequences do not differ consistently and the observed minor variation (within 0.3%) does not correlate with either morphology or locality^13^. The lack of a clear hiatus in morphology and mitochondrial DNA sequence questions their rank as distinct biological species.

To better understand the evolution and speciation of North American *Calycopis*, we obtained complete genomes of 3 *C. cecrops* and 5 *C. isobeon* specimens, including a nominotypical specimen from Costa Rica. In contrast to the mitochondrial DNA results and despite morphological closeness, their nuclear genomes revealed a large divergence between the two species (1.4% in the coding regions). However, the divergence between *C. isobeon* and *C. cecrops* is attributed to a relatively small fraction of genes: only 22% of gene sequences can clearly separate the two species and only 10% can also distinguish them by the protein sequences they encode (divergence hotspots). These divergence hotspots and their function overlap significantly with those hotspots identified in other pairs of sister species, such as *Pterourus glaucus* and *Pterourus canadensis*^1^. The most significantly enriched biological processes assigned to speciation hotspots in both *Calycopis* and *Pterourus* is the circadian clock system, with all the four central components, CLOCK, CYCLE, PERIOD and TIMELESS being considerably more divergent between species than within species. The divergence in these proteins may cause Dobzhansky-Muller (DM) hybrid incompatibility^14^, because the circadian clock components of one species may not be fully compatible with ones of another species. Divergence in circadian clock system could be a common speciation mechanism in butterflies, particularly for sister species mainly confined to different latitudes. Finally, we identified 98 nuclear barcodes that could unambiguously identify the two species. This work lays the foundation for population studies of *Calycopis*, including the studies of possible hybridization between *C. isobeon* and *C. cecrops* in the zone of sympatry.

## Results and Discussion

### Genome assembly, annotation, and comparison to other Lepidoptera genomes

We assembled a 729 Mb reference genome of *Calycopis cecrops* (*Cce*), which is the largest among currently sequenced Lepidoptera genomes^1^,^4^,^5^,^6^,^15^,^16^,^17^,^18^,^19^. This genome size is consistent with those (760 Mb and 660 Mb, respectively) estimated by flow cytometry (supplemental Fig. S1) and frequency of k-mers in the sequencing reads (supplemental Fig. S2). The scaffold N50 of *Cce* genome assembly is 233 kb, comparable to many other published Lepidoptera genomes. The genome assembly is better than many other Lepidoptera genomes in terms of completeness measured by the presence of Core Eukaryotic Genes Mapping Approach (CEGMA) genes (supplemental Table S1B)^20^, cytoplasmic ribosomal proteins and independently assembled transcripts (Table 1). The genome sequence has been deposited at DDBJ/EMBL/GenBank under the accession LUGF00000000. The version described in this paper is version LUGF01000000. In addition, the main results from genome assembly, annotation and analysis can be downloaded at http://prodata.swmed.edu/LepDB/.

We assembled the transcriptome of *Cce* using another specimen from the same locality. Based on the transcriptome, homologs from other Lepidoptera and *Drosophila melanogaster*, *de novo* gene predictions, and repeat identification (supplemental Table S2B), we predicted 16,456 protein-coding genes in the *Cce* genome (supplemental Table S2C). 80% of these genes are likely expressed in the adult, as they fully or partially overlap with the transcripts. We annotated the putative functions for 14,379 protein-coding genes (supplemental Table S2D). Although the genome size of *Cce* is 2–3 times that for other Lepidoptera genomes, the number of proteins encoded by the genome is comparable to other Lepidoptera. This discrepancy indicates the dramatic increase in size of *Cce* genome arises from expansion in the non-coding regions and transposons in the genome.

Comparison of the protein sets from Lepidoptera species revealed expansions of many gene families in *Calycopis* (supplemental Table S3). The expanded families include a variety of oxidoreductases, and thus they show the most significant functional enrichment in oxidation-reduction process (GO:0055114). For instance, several families of Cytochrome P450 underwent expansion. The expansion of oxidoreductases may be related to production of pigments, synthesis of pheromones, and the ability for *Calycopis* to feed on detritus and fungi. Detritus and fungi contain various xenobiotic and toxins that might be unpalatable for most other butterfly caterpillars, and oxidation-reduction processes, such as many cytochrome P450 monooxygenases can oxidize the toxic compounds to less toxic products^21^, offering an efficient way of detoxification. Calycopis is somewhat unusual among USA butterflies that it does not naturally feed on green leaves. While in captivity *C. cecrops* caterpillars can feed to pupation on green leaves of mulberry trees (*Morus*, unpublished observations), staghorn sumac (*Rhus typhina*) and wax myrtle (*Myrica cerifera*)^22^. In nature, *C. cecrops* and *C. isobeon* are likely to be exclusively detritivores in leaf litter (caterpillars are dark-brown) and rarely, if ever, are found on live leaves of plants^23^. Additionally, in captivity, the caterpillars readily accepted moldy bread and rotting leaves from many plant families (sumac, elm, oaks, unpublished observations).

Similarly, another family of detoxification enzymes, Glutathione S-transferases also underwent expansion in *Calycopis*. Several families of proteins related to digestion, such as protease and salivary secreted peptides also underwent expansion, which possibly accounts for its polyphagy. Many new World Eumaeini species are exceedingly polyphagous^24^,^25^, and they may show similar gene expansion as we observed in *Calycopis*. Analogously, Edger *et al*. found that gene duplications were involved in major larval food plant shifts in Pieridae^26^.

### Eight genomes of *Calycopis*

**Figure 2:**
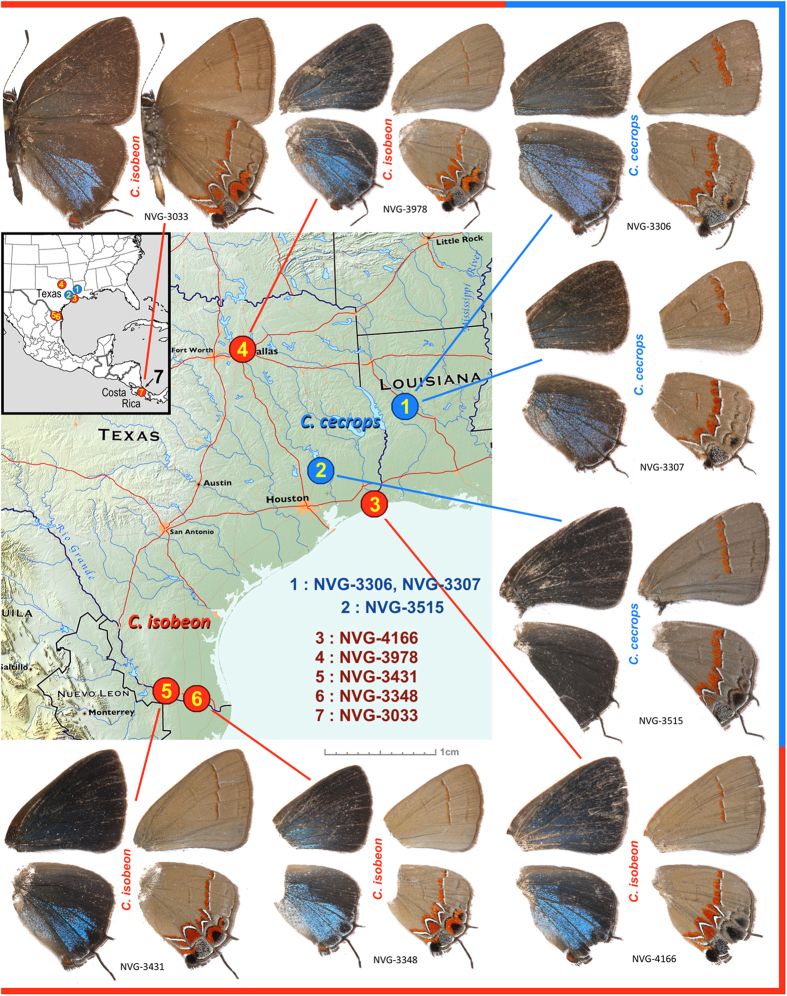
Sequenced *Calycopis* specimens and their localities. *C. cecrops* specimens are labeled in blue and *C. isobeon* are labeled in red. Dorsal and ventral views of each specimen are shown on the left and right, respectively. Only wings are shown for specimens whose bodies were used for DNA extraction. Map services and data available from U.S. Geological Survey, National Geospatial Program (USGS home page http://www.usgs.gov).

In addition to the reference genome of *C. cecrops* from western Louisiana, we sequenced the complete genomes of seven *Calycopis* specimens and mapped the reads to the reference. Two specimens were *C. cecrops* from the same locality and southeastern Texas, three were *C. isobeon* from several localities in Texas, and the last was nominotypical *C. isobeon* from Costa Rica (Fig. 2). Most of these were specimens collected in the field in 2015 and preserved in *RNAlater* solution. The only exception is the Costa Rican specimen, which was collected in 1986, pinned and stored dry in Smithsonian collection in Washington DC. The coverage by the reads and the completeness of these genomes are in Table 2. The sequencing reads for all the specimens cover the genome 12–17 times.

About 98% of coding regions in the reference genome can be mapped by reads from each specimen, and this ratio is higher for the two specimens that were used to assemble the reference genome. The fractions of non-coding region that can be mapped differ significantly (p = 0.00023) between specimens. Reads from specimens of the same species (*C. cecrops*) as the reference genome can map to 88% of positions in the reference genome while reads from the specimens of a different species (*C. isobeon*) can map to only 83% of positions. This result indicates substantial differences between the two species in the non-coding region. Interestingly, the coverage of the reference genome by reads from the 30 year old dry specimen (NVG-3033) in the museum is not much worse than fresh specimens (96% for coding region and 82.5% for non-coding region). This coverage might be further increased by increasing the depth of sequencing, proving the possibility of using the extant material in the museums for population and comparative genomics studies.

We identified SNPs in these genomes compared to the reference genome using Genome Analysis Toolkit (GATK)^27^. The fraction of heterozygous positions in each specimen varies from 1.2% to 2.0%, and the heterozygosity level is negatively related to (P = 0.01) the latitude of the locality from which the specimen was collected (supplemental Fig. S3). Localities at lower latitudes with warmer climates typically harbor larger populations and a larger number of broods per year, which presumably increases genetic variation. In addition, the southern species may have a higher chance to receive gene flow from other *Calycopis* species in the Neotropics, which also could diversify its gene pool. In all genomes, the percentages of SNPs in the coding regions (0.56% ~ 1.04%) is much lower than that for the non-coding regions (1.18% ~ 1.99%), which is likely due to the potential deleterious effect of SNPs in the coding regions.

### Phylogeny of Lepidoptera

**Figure 3:**
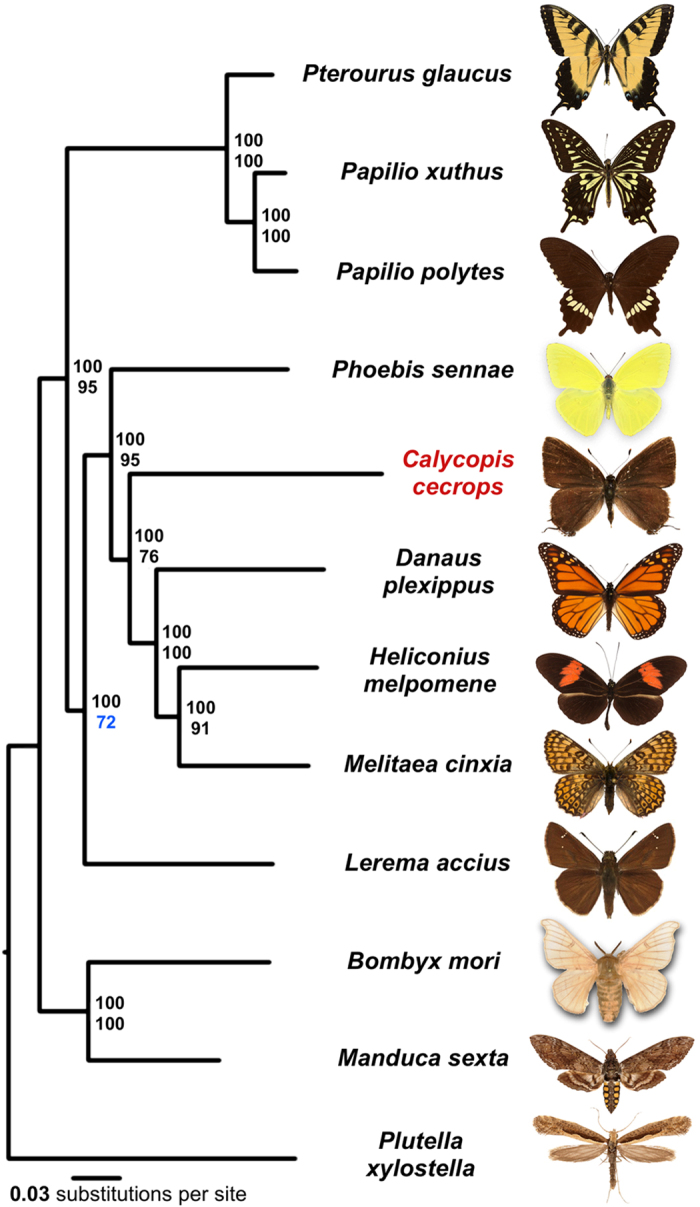
Phylogenetic tree of the Lepidoptera species with complete genome sequences. Majority-rule consensus tree of the maximal likelihood trees constructed by RAxML on the concatenated alignment of universal single-copy orthologous proteins. Numbers by the nodes refer to bootstrap percentages. The numbers above are obtained from the complete alignment, the number below are obtained on 1% of the dataset.

We identified orthologous proteins encoded by 12 Lepidoptera genomes (*Plutella xylostella*, *Bombyx mori*, *Manduca sexta*, *Lerema accius*, *Pterourus glaucus*, *Papilio polytes*, *Papilio xuthus*, *Phoebis sennae*, *Melitaea cinxia*, *Heliconius melpomene*, *Danaus plexippus*, and *Calycopis cecrops*) and detected 4951 universal orthologous groups, from which 1894 consist of a single-copy gene in each of the species. A phylogenetic tree built from the concatenated alignment of the single-copy orthologs using RAxML places *Calycopis* as the sister to the Nymphalidae clade (Fig. 3). This placement agrees with the previously published results^28^. In addition, our analysis places Papilionidae as a sister to all other butterflies, including skippers (Hesperiidae). Such placement contradicts morphology-based phylogeny, but is reproduced in all maximum-likelihood and Bayesian trees published recently^8^,^29^.

All nodes received 100% bootstrap support when the alignment of all single-copy orthologs was used. However, since bootstrap only measures internal consistency of phylogenetic signal in the alignment, very large datasets will almost always result in 100% support, even if the tree is incorrect and biased by various effects such as nucleotide composition bias and long branch attraction. To find the weakest nodes, we reduced the amount of data by randomly splitting the concatenated alignment of all single-copy orthologs into 100 alignments (about 3200 positions in each alignment). The consensus tree based on these alignments revealed that the node referring to relative position of skippers and swallowtails shows the lowest support (72%) compared to other nodes, and their evolutionary history remains to be further investigated when better taxon sampling by complete genomes is achieved. The placement of *Calycopis* in the tree is also less strongly supported (76%) compared to other taxa, which may be related to its elevated evolutionary rate (*C. cecrops* is a long branch in the phylogenetic tree shown as Fig. 3) compared to other species.

### Discordant evolution of nuclear and mitochondrial genes

**Figure 4:**
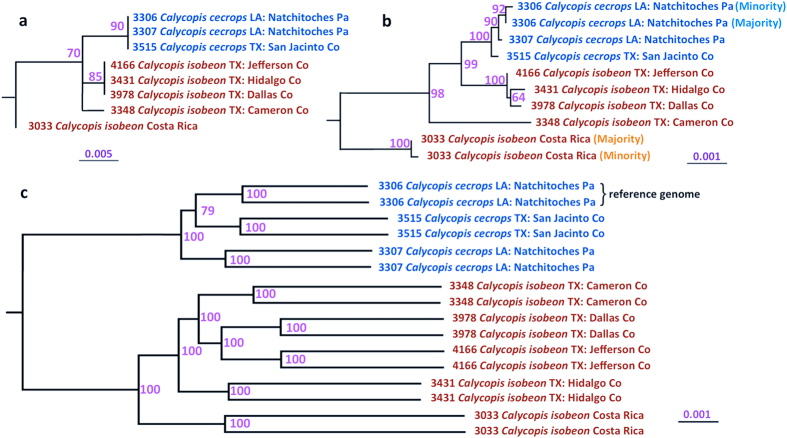
Incongruence between phylogeny inferred from mitochondrial and genomic DNA. Trees obtained from alignments of (**a**) COI mitochondrial DNA barcode, (**b**) mitochondrial genes, and (**c**) nuclear genes. Specimen numbers, species names and localities are given. Two branches in nuclear trees corresponding to the same specimen refer to father and mother copies. Mitochondria of specimen 3033 and 3306 revealed two distinct types. Numbers by the nodes refer to bootstrap percentages.

To study speciation in *Calycopis*, we compared the nuclear and mitochondrial genomes of 8 specimens. The analysis based on sequences of COI barcodes and the complete mitochondrial genomes failed to confidently separate *Calycopis* species: *C. isobeon* is not monophyletic in the phylogenetic trees built from mitochondrial sequences (Fig. 4a,b and supplemental Fig. S4). The nominotypical *C. isobeon* from Costa Rica is separated from both species in the US. Even among US specimens, NVG-3348 does not cluster with other US *C. isobeon* specimens in a tree from all mitochondrial genes (Fig. 4b). Thus, mitochondrial DNA suggests that either *C. cecrops* may be conspecific with *C. isobeon*, or populations in the US currently assigned to *C. isobeon* may be neither of the two species.

In contrast, the two *Calycopis* species are clearly separated in a tree based on nuclear genomes, and both species are monophyletic (Fig. 4c and supplemental Fig. S4) based on the concatenated alignment of nuclear genes (16,306 genes, and 21,502,436 base pairs). *C. cecrops* and *C. isobeon* diverge in about 1.3% ~ 1.5% of nucleotides in the protein-coding regions, and the branch length between the two species in the phylogenetic tree is 0.7%. While the nominotypical specimen from Costa Rica is placed as sister to *C. isobeon* specimens from the US, its evolutionary distance to the *C. isobeon* specimens from the US is much smaller than the divergence between the two *Calycopis* species. The confident separation of *C. cecrops* and *C. isobeon* by nuclear genomes and relative closeness of *C. isobeon* genomes from Texas and Costa Rica despite vast geographic distance (> 2000 km) reaffirms the status of the two *Calycopis* as distinct species.

Failure to distinguish these two species using the mitochondrial genomes cautions about the sole use of mitochondrial DNA for species classification. Incongruence between trees built from nuclear and mitochondrial genes can be explained by introgression of mitochondria at a time more recent than the split of these two species. At short evolutionary distances the exchange of genetic material between different species is still possible, and introgression was observed in several lineages^5^,^30^,^31^. Introgressed mitochondria may become fixed by population bottlenecks or favorable selection pressure. However, according to the Haldane’s rule, the heterogametic sex (i.e., females in Lepidoptera) in hybrids is likely to be less fit, which is a barrier to mitochondrial DNA flow. Thus, alternatively, it is possible that mitochondria of *C. cecrops* and *C. isobeon* have not diverged sufficiently to distinguish the two species, similar to 77% of nuclear genes that do not show strong interspecies divergence. Although mitochondrial DNA usually tends to evolve faster than nuclear DNA^32^, its evolutionary rate varies a lot among lineages and could be slower than the rate for nuclear DNA in some species^33^,^34^,^35^.

### Molecular processes differentiating *C. cecrops* and *C. isobeon*

*C. cecrops* and *C. isobeon* are well-separated based on the whole-genome data. Nevertheless, the two species have not clearly diverged in most individual genes, and only 22% of genes can confidently (bootstrap >= 95%) distinguish them based on the DNA sequence. To further investigate the possible phenotypic consequences caused by genetic divergence between the two *Calycopis* species, we focused on the non-synonymous mutations. We identified genes that can confidently distinguish (separate the two taxa into clades with no less than 95% bootstrap support) the two species both by their sequences and by the sequences of proteins they encode.

We identified 1653 (10%) such proteins (supplemental Table S4A); however, about half of these proteins are not conserved in either species. These proteins are probably intrinsically more tolerant to mutations and therefore they can undergo fast evolution, and removal of them from the list results in 780 proteins that can clearly distinguish the two species (supplemental Table S4B) and yet are conserved within at least one species. Some of these proteins may be directly related to the reproductive barrier of the two species: they could cause Dobzhansky-Muller hybrid incompatibility as the proteins from *C. cecrops* may not work well with those from *C. isobeon* when functioning in the same pathway. We term these proteins speciation hotspots.

**Figure 5:**
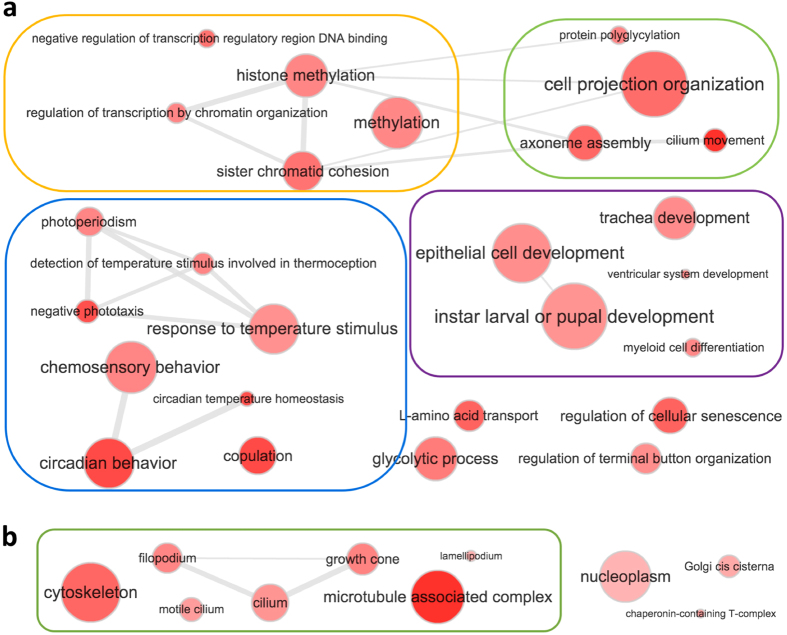
Significantly enriched GO terms associated with speciation hotspots of *Calycopis*. (**a**) GO terms in the category of biological process and (**b**) GO terms in the category of cellular components. Each red dot represents one GO term as marked in the figure, and grey lines connect GO terms that are related and frequently associated with the same proteins. The darker dot color corresponds to a higher level of significance and the dot size is positively correlated to the number of *Drosophila* proteins associated with this GO term. GO terms inside the orange, green, blue, purple and red boxes are generally related to transcription regulation, cytoskeleton, response to light and circadian behavior, and development; respectively.

**Figure 6:**
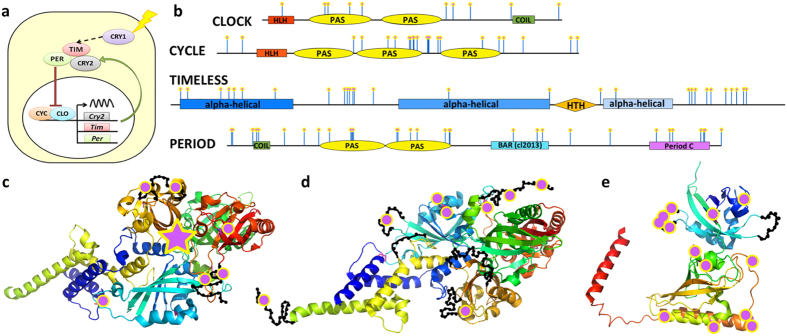
Main components of circadian clock system are divergent between *Calycopis* species. (**a**) Circadian clock system (CRY: cryptochrome proteins; CLO: CLOCK; CYC: CYCLE; PER: PERIOD; TIM: TIMELESS). (**b**) Domain diagram of CLOCK, CYCLE, PERIOD, and TIMELESS. Positions that are conserved within but differ between species are marked by pink dots on blue stems. (**c**,**d**) Map of interspecific mutations on the spatial structure template (PDB id: 4F3L) of the CLOCK/CYCLE complex. The mutations are marked by magenta dots and the approximate position of disordered loops is shown as black beads on threads. The magenta star in (**c**) indicates the position of an inserted domain rich in interspecific mutations that is not present in the structural template. (**e**) Map of interspecific mutations on the spatial structure template (PDB id: 4F3L) of the protein PERIOD (PDB id: 3RTY_A).

GO-term analysis of these speciation hotspots revealed prevalence of the circadian clock system, mating behavior, transcription regulation, development and cytoskeleton (Fig. 5, supplemental Fig. S5 and Table S4C). The four main components of the circadian clock system, i.e., CLOCK, CYCLE, PERIOD and TIMELESS (Fig. 6a) are all among speciation hotspots. Mapping amino acid differences between species to the sequences (Fig. 6b) and 3D structures (Fig. 6c–e) shows that these interspecific mutations cluster on the surface of these proteins. The surface clustering of mutations suggest that they likely modify interactions between circadian clock proteins and other regulators. This altered surface may cause the circadian clock components from one species to be incompatible with those from another species, leading to post-zygotic reproductive barrier. The circadian clock proteins also play an important role in regulating the timing of mating and thus affect the mating behavior^36^,^37^. Therefore, divergence in the circadian behavior may lead to differences in mating behavior and contribute to a pre-zygotic reproductive barrier as well.

Similarly, the divergence in many cytoskeleton components that interact with each other could contribute to a post-zygotic reproductive barrier, although these may not cause obvious morphological divergence. Differences in transcriptional factors might be another easy source of hybrid incompatibility because transcription factors tend to participate in multi-protein complexes as well as directly interact with DNA, especially the non-coding regions that could evolve rapidly. In addition, transcriptional factors and proteins related to development may have a profound impact on the physiology of an organism, driving the divergence and speciation.

**Figure 7:**
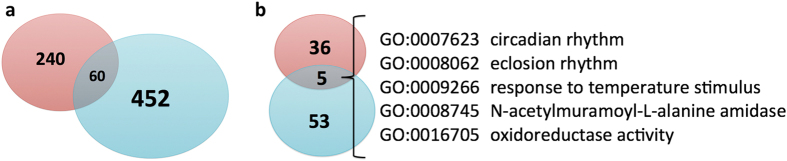
Significant overlap between the speciation hotspots in *Calycopis* and *Papilio*. (**a**) Venn diagram of speciation hotspots for *Calycopis* (blue) and *Pterourus* (red), and they overlap significantly (p = 5.86e-12). (**b**) Venn diagram for the enriched GO-terms associated with the speciation hotspots for *Calycopis* (blue) and *Papilio* (red), and they overlap significantly (p = 3.73e-6). The five GO-terms that are shared in both genera are listed.

The speciation hotspots for *Calycopis* species overlap significantly (P = 5.86e-12, Fig. 7a) with those we previously identified for swallowtail butterflies (*Pterourus glaucus* and *Pterourus canadensis*)^1^. The enriched GO terms associated with the *Calycopis* speciation hotspots also overlap significantly (p = 3.73e-6, Fig. 7b) with those for *Pterourus*, indicating some common mechanisms for both speciation events. The most significantly enriched GO terms (supplemental Fig. S6 and Table 4D) associated with the speciation hotspots in both genera are related to circadian clock and copulation. The four main components of the circadian clock system, CLOCK, CYCLE, TIMELESS and PERIOD are the speciation hotspots in both cases. To the extent that *P. canadensis* (single brood) / *P. glaucus* (multiple broods) and *C. cecrops* (diapauses during freezing weather) / *C. isobeon* (may not deal with hard freezes well) are “latitudinal species pairs”, the divergence in the circadian clock system could be a general mechanism of adaptive speciation when the two sister species are adapted to different latitudes with somewhat different climates and sunlight patterns^38^,^39^.

### Nuclear DNA markers to identify *C. cecrops* and *C. isobeon*

**Figure 8:**
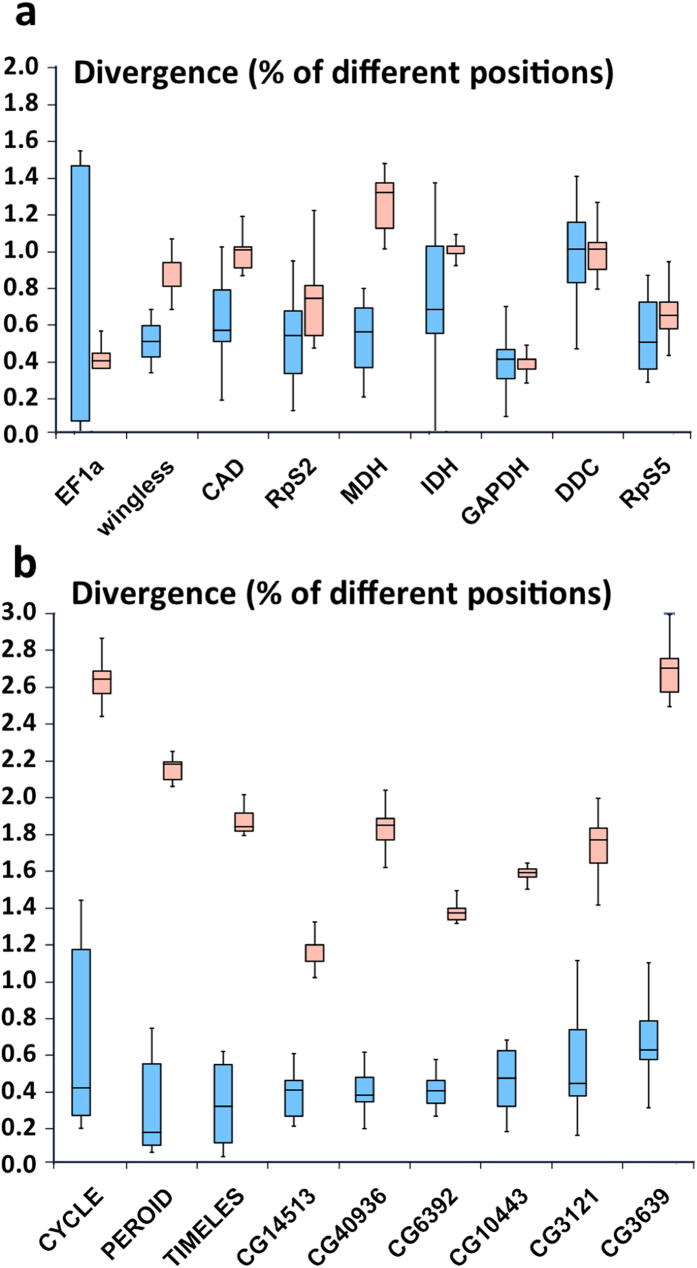
Divergence of selected nuclear gene markers within (blue) and between (red) *Calycopis* species. (**a**) Nuclear genes commonly used in phylogenetic analysis of Lepidoptera (general nuclear markers). (**b**) Examples of nuclear genes that discriminate best between the taxa based on this study. See supplemental Table S5 for information about these markers.

The failure to identify *C. cecrops* and *C. isobeon* based on mitochondrial genomes calls for investigation of nuclear DNA markers for their identification. The two species cannot be clearly identified using nuclear markers previously selected for butterfly phylogeny (Fig. 8). This situation is similar to that of *Pterourus glaucus* and *P. canadensis* (supplemental Fig. S7). Out of 16306 nuclear genes with sufficient coverage for analysis, 1232 genes’ minimal interspecific divergence at both DNA and protein level is at least 0.1% higher than the maximal intraspecific divergence. 148 of these genes (supplemental Table 5A) are associated with enriched GO terms associated with the divergence hotspots. They likely participate in the biological processes that have diverged between the two taxa and might be used as nuclear markers to identify the two species. Several examples of these nuclear markers, such as the circadian clock proteins CYCLE, PERIOD, and TIMELESS are illustrated in Fig. 8. We focused on the exons of these 148 genes and identified 98 exons (supplemental Table S5B) that might be suitable for species identification by PCR amplification of this region. These exons are conserved at the N- and C-termini for primer design and yet show high interspecific divergence in the middle.

### Possible evolutionary scenarios in the cecrops group of *Calycopis*

The cecrops group of *Calycopis* is uniquely characterized by the shape of the uncus in lateral view^11^,^12^ and includes two species: *C. cecrops* and *C. isobeon*. They are very similar morphologically without apparent differences in genitalia, and some specimens are nearly impossible to identify with confidence by wing patterns. COI barcodes and mitochondrial DNA do not distinguish them either. Interestingly, nominotypical *C. isobeon* from Costa Rica differs from both *Calycopis* species in the US by COI barcodes and possesses a shorter penis, although penis length is clinal from north to south. Four taxonomic scenarios are possible. First, is the “lumper” hypothesis according to which *C. isobeon* is the same species as *C. cecrops*. Second, *C. isobeon* is a distinct Central American species, and isobeon-like populations in the US are *C. cecrops*. Third, the traditional definition of the two species holds, but USA populations of *C. isobeon* either experienced introgression of mitochondrial DNA from *C. cecrops*, or both species largely share mitochondrial polymorphism across their ranges. And the last is the “splitter” hypothesis that South Texas populations formerly referred to as *C. isobeon* are a new species, distinct from both *C. cecrops* and *C. isobeon*.

Complete genome sequencing of individuals from both species revealed unexpectedly large divergence between *C. cecrops* and *C. isobeon* in nuclear genes. This divergence appears more prominent than that between the two swallowtails species *Pterourus canadensis* and *P. glaucus*. The two *Calycopis* species show significant divergence in cell skeleton proteins and circadian clock proteins. These proteins may not cause apparent differences in morphology. However, the fact that the two species have diverged in proteins in the same complex/pathway may cause DM hybrid incompatibility and contribute to the reproductive barrier. In particular, the divergence hotspots for the two *Calycopis* species and two *Pterourus* species significantly overlap, with circadian clock system being the most prominently diverged in both cases. This data suggests the existence of common speciation mechanisms, and the divergence in circadian clock system as the two species adapts to different latitudes and climates might be repeatedly observed.

Our results offer best support to the third hypothesis stated above, i.e., the traditional definition of the two *Calycopis* species, with *C. cecrops* being the eastern USA taxon, and *C. isobeon* being mostly Texan, distributed southwards to Costa Rica, which is its type locality, and Panama. Although our results highlight the genomic differences between *C. cecrops* and *C. isobeon*, the history of their speciation, i.e. if it occurs with primary or secondary intergradation remains to be investigated^40^. It is also clear that *C. isobeon* populations in Costa Rica and Texas diverged less from each other than either diverged from *C. cecrops* (Fig. 4). However, we cannot completely rule out the possibility that the two *C. isobeon* populations (from the US and Costa Rica) could be incipient species, and broader sampling of *C. isobeon* specimens throughout its range is needed to better understand the population structure of *C. isobeon*.

Some individuals in the zone of sympatry of *C. cecrops* and *C. isobeon* appear intermediate in wing patterns and may be hybrids. Two widespread species hybridizing in a relatively narrow zone where they meet is a common biological phenomenon^40^. In butterflies, sister species from the genus *Anartia* may be the best studied case in which it was shown that a hybridization zone is maintained by F2 and backcross incompatibility^41^. More generally, narrow hybridization zones may be maintained for tens of thousands of years without compromising either species, as shown in beetle fossils preserved in peat^42^.

## Conclusions

We report eight genomes of Hairstreaks, three of *Calycopis cecrops* and five of *Calycopis isobeon*. Being the first sequenced genomes from the family Lycaenidae, they offer rich datasets for comparative population genomics and phylogenetic studies of Lepidoptera. The assembled genome size of *Calycopis* is 729 Mb, representing the largest Lepidoptera genome sequenced to date, which coupled with their high heterozygosity (1.2%) represented a challenging problem in invertebrate genomics. Comparative analyses of *Calycopis* genomes revealed a significant discordance in the evolution of nuclear and mitochondrial genomes. While mitochondrial trees show very low divergence and render *C. isobeon* polyphyletic, nuclear genomes demonstrate a deep split between the species, both of which are monophyletic. This work provides another example of problems in relying exclusively on mitochondrial genomes to derive phylogenetic and taxonomic conclusions.

We identified genes that can separate the two *Calycopis* species (speciation hotspots) and showed that theydiverged from each other in proteins related to circadian clock system, mating behavior, transcription regulation, development and cytoskeleton. The presence of multiple divergent proteins that function together in these pathways could cause a certain level of reproductive barrier due to DM hybrid incompatibility. The speciation hotspots and their functions significantly overlap with those we previously identified for *Pterourus*, suggesting common speciation mechanisms. Finally, we identified about 100 nuclear markers to distinguish these two species, such as coding regions from circadian clock proteins CYCLE, PERIOD and TIMELESS. These markers should be helpful for species identification in a large-scale study of *Calycopis*.

## Methods

### Library preparation and sequencing

We removed and preserved the wings and genitalia of seven freshly caught *Calycopis* specimens (three *C. cecrops*: NVG-3306 and NVG-3307, females, Louisiana: Natchitoches Parish, Kisatchie National Forest, 31.524582, −93.098485, 12-Apr-2015; NVG-3515, male, Texas: San Jacinto Co., Sam Houston National Forest, 30.50644, −95.08783, 7-Jun-2015; and four *C. isobeon*: NVG-3978, female, Texas: Dallas Co., Dallas, Norbuck Park, 32.85531, −96.71484, 8-Jul-2015; NVG-4166, female, Texas, Jefferson Co. southeast of Sabine Pass, 29.72914, −93.87508, 18-Jul-2015; NVG-3431, male, Texas: Hidalgo Co., Penitas, 26.22615, −98.43653, 30-May-2015; NVG-3348, female, Texas: Cameron Co, 1.4 mi south of Santa Maria, 26.05681, −97.84284, 23-May-2015), and the rest of the bodies were stored in *RNAlater* solution. Wings and genitalia of these specimens will be deposited in the National Museum of Natural History, Smithsonian Institution, Washington, DC, USA (USNM). Abdomen of *C. isobeon* male NVG-3033 from Costa Rica: San Jose, Patarra 9.8833, −84.0333, 24-Jun-1980, leg. G. B. Small, in the USNM collection, was taken from the dry specimen, DNA was extracted and genitalia were dissected (NVG141101-64).

We used specimens NVG-3306 and NVG-3307 to assemble the reference genome. We extracted genomic DNA from them with the ChargeSwitch gDNA mini tissue kit. 250 bp and 500 bp paired-end libraries were prepared using genomic DNA from specimen NVG-3306 with enzymes from NEBNext Modules and following the Illumina TruSeq DNA sample preparation guide. 2 kb, 6 kb and 15 kb mate pair libraries were prepared using genomic DNA from both NVG-3306 and NVG-3307 with a protocol similar to previously published Cre-Lox-based method 43. For the 250 bp, 500 bp, 2 kbp, 6 kbp and 15 kbp libraries, approximately 500 ng, 500 ng, 1.5 μg, 3 μg and 5 μg of DNA were used, respectively. We quantified the amount of DNA from all the libraries with the KAPA Library Quantification Kit, and mixed 250 bp, 500 bp, 2 kbp, 6 kbp, 15 kbp libraries at relative molar concentration 40:20:8:4:3. The mixed library was sequenced for 150 bp at both ends using two lanes of Illumina HiSeq2500 at UT Southwestern Medical Center genomics core facility.

Part of specimen NVG-3306 was used to extract RNA using QIAGEN RNeasy Mini Kit. We further isolated mRNA using NEBNext Poly(A) mRNA Magnetic Isolation Module and RNA-seq library was prepared with NEBNext Ultra Directional RNA Library Prep Kit for Illumina following manufactory’s protocol. The RNA-seq library was sequenced for 150 bp from both ends using 1/6 of an Illumina lane.

We prepared paired-end libraries for NVG-3307 and the other six specimens to map to the reference genome. We extracted genomic DNA as described above and used about 500 ng genomic DNA to prepare paired-end libraries. For fresh specimens, we selected the insert size to be about 400 bp. Since genomic DNA was largely degraded (fragment size <1000 bp) for the dry specimen NVG-3033, we did not fragment the DNA or perform size selection so that the coverage of the genome is maximized. These paired-end libraries were mixed at equal ratio and sequenced for 150 bp at both ends using 1.2 Illumina lanes to get about 12-fold coverage for each specimen. The sequencing reads for all the specimens have been deposited in NCBI SRA database under accession SRP071639.

### Genome and transcriptome assembly

We removed sequencing reads that did not pass the purity filter and classified the pass-filter reads according to their TruSeq adapter indices to get individual sequencing libraries. Mate pair libraries were processed by the Delox script^43^ to remove the loxP sequences and to separate true mate pair reads from paired-end reads. All reads were processed by mirabait^44^ to remove contamination from the TruSeq adapters, an in-house script to remove low quality portions (quality score <20) at the ends of both reads, JELLYFISH^45^ to obtain k-mer frequencies in all the libraries, and QUAKE^46^ to correct sequencing errors. The data processing resulted in seven libraries that were supplied to Platanus^47^ for genome assembly: 250 bp and 500 bp paired-end libraries, 2 kbp, 6kbp, 15k bp true mate pair libraries, a library containing all the paired-end reads from the mate pair libraries, and a single-end library containing all reads whose pairs were removed in the process (supplemental Table S2A).

We mapped these reads to the initial assembly with Bowtie2^48^ and calculated the coverage of each scaffold with the help of SAMtools^49^. Many short scaffolds in the assembly showed coverage that was about half of the expected value; they likely came from highly heterozygous regions that were not merged to the equivalent segments in the homologous chromosomes. We removed them if they could be fully aligned to another significantly less covered region (coverage > 90% and uncovered region < 500 bp) in a longer scaffold with high sequence identity (>95%). Similar problems occurred in the *Heliconius melpomene*, *Pterourus glaucus* and *Lerema accius* genome projects, and similar strategies were used to improve the assemblies 1,5,8.

The RNA-seq reads were processed using a similar procedure as the genomic DNA reads to remove contamination from TruSeq adapters and the low quality portion of the reads. Afterwards, we applied three methods to assemble the transcriptomes: (1) *de novo* assembly by Trinity^50^, (2) reference-based assembly by TopHat^51^ (v2.0.10) and Cufflinks^52^ (v2.2.1), and (3) reference-guided assembly by Trinity. The results from all three methods were then integrated by Program to Assemble Spliced Alignment (PASA)^53^.

### Identification of repeats and gene annotation

Two approaches were used to identify repeats in the genome: the RepeatModeler^54^ pipeline and in-house scripts that extracted regions with coverage 4 times higher than expected. These repeats were submitted to the CENSOR^55^ server to assign them to the repeat classification hierarchy. The species-specific repeat library and all repeats classified in RepBase^56^ (V18.12) were used to mask repeats in the genome by RepeatMasker^57^.

We obtained two sets of transcript-based annotations from two pipelines: TopHat followed by Cufflinks and Trinity followed by PASA. In addition, we obtained nine sets of homology-based annotations by aligning protein sets from *Drosophila melanogaster*^58^ and eight published Lepidoptera genomes (*Plutella xylostella*, *Bombyx mori*, *Lerema accius*, *Papilio polytes*, *Papilio glaucus*, *Papilio xuthus*, *Heliconius melpomene*, and *Danaus plexippus*) to the *Calycopis cecrops* genome with exonerate^59^. Proteins from Insects in the entire UniRef90^60^ database were used to generate another set of gene predictions by genblastG^61^. We manually curated and selected 897 confident gene models by integrating the evidence from transcripts and homologs to train *de novo* gene predictors: AUGUSTUS^62^, SNAP^63^ and GlimmerHMM^64^. These trained predictors, the self-trained Genemark^65^ and a consensus-based pipeline Maker^66^, were used to generate another five sets of gene models. Transcript-based and homology-based annotations were supplied to AUGUSTUS, SNAP and Maker to boost their performance. In total, we generated 16 sets of gene predictions and integrated them with EvidenceModeller^53^ to generate the final gene models.

We predicted the function of *Cce* proteins by transferring annotations and GO-terms from the closest BLAST^67^ hits (E-value <10^−5^) in both the Swissprot^68^ database and Flybase^69^. Finally, we performed InterproScan^70^ to identify conserved protein domains and functional motifs, to predict coiled coils, transmembrane helices and signal peptides, to detect homologous 3D structures, to assign proteins to protein families and to map them to metabolic pathways.

### Identification of orthologous proteins, gene expansion, and phylogenetic tree reconstruction

We identified the orthologous groups from all 12 Lepidoptera genomes using OrthoMCL^71^. If two OrthoMCL-defined orthologous groups overlapped in the *Drosophila* proteins that they mapped to, we merged them into one family. The function of each family was annotated using GO terms. GO terms that are associated with any gene in a family are considered to be associated with this family. The total number and total length of proteins in a family were used to identify expanded gene families in *Calycopis*. If the total number and length of *Calycopis* proteins in a family are more than 1.5 times of the average number and length across other Lepidoptera species, we consider this protein family to have undergone expansion in *Calycopis*. The enrichment GO terms associated with these expanded families were identified using binomial tests: m = the number of expanded gene families that were associated with this GO term, N = number of expanded gene families, p = the probability for this GO term to be associated with any gene family.

1894 orthologous groups consisted of single-copy genes from every species, and they were used for phylogenetic analysis. An alignment was built for each universal single-copy orthologous group using both global sequence aligner MAFFT^72^ and local sequence aligner BLASTP. Positions that were consistently aligned by both aligners were extracted from each individual alignment and concatenated to obtain an alignment containing 319,945 positions. The concatenated alignment was used to obtain a phylogenetic tree using RAxML^73^. Bootstrap resampling of the aligned positions was performed to assign the confidence level of each node in the tree. In addition, in order to detect the weakest nodes in the tree, we reduced the amount of data by randomly splitting the concatenated alignment into 100 alignments (about 3,200 positions in each alignment) and applied RAxML to each alignment. We obtained a 50% majority rule consensus tree and assigned confidence level to each node based on the percent of individual trees supporting this node.

### Assembly and annotation of mitochondrial genomes

The mitogenomes of several closely related species, including *Coreanna raphaelis*^74^, *Japonica lutea*^75^, and *Protantigius superans*^76^ were used as reference. Based on these mitogenomes, we applied mitochondrial baiting and iterative mapping (MITObim) v1.6^77^ software to extract the sequencing reads of the mitogenome in the paired-end libraries for specimen NVG-3306. We used JELLYFISH to obtain the frequencies of 15-mers in these reads, and applied QUAKE to correct errors in 15-mers with frequencies lower than 1,000 and excluded reads containing low-frequency 15-mers after error correction. We assembled the error-corrected reads into contigs *de novo* with Platanus. We manually selected the contig corresponding to the mitogenome (the longest one with highest coverage), and extended its sequence manually by baiting in the sequencing reads to obtain a complete circular DNA. In addition, by aligning the protein coding sequences from the mitogenomes of closely related species mentioned above to the *Cce* mitogenome, we annotated the 13 protein coding genes.

### Obtaining the genomes of eight *Calycopis* specimens and phylogenetic analysis

We mapped the sequencing reads of all 8 *Calycopis* specimens to the reference genome (including the mitochondrial genome) using BWA^78^ and detected SNPs using the Genome Analysis Toolkit (GATK)^79^. We deduced the genomic sequences for each specimen based on the result of GATK. We used two sequences to represent the paternal and maternal DNA in each specimen. For heterozygous positions, each possible nucleotide was randomly assigned to either paternal or maternal DNA. Based on the gene annotation of the reference genome, we further deduced the protein-coding sequences of genes in each specimen.

16,306 nuclear and 13 mitochondrial genes of *Calycopis* were no less than 50% covered in at least two *C. cecrops* and two *C. isobeon* specimens, and they were used for the comparative analysis. Alignments of these genes were derived from the mapping results to the reference genome using in-house scripts. We concatenated the alignments of the 16,306 nuclear genes and 13 mitochondrial genes, respectively to obtain an alignment of nuclear genes and an alignment of mitochondrial genes. The two alignments were used to build both neighbor-joining trees with PHYLIP^80^ based on the percentage of different positions between specimens and maximal-likelihood trees with RAxML (model: GTRGAMMA). Bootstrap resampling was performed to assign confidence levels for nodes in the maximal-likelihood tree.

### Identification of speciation hotspots

We identified speciation hotspots using the alignments of individual genes that were derived from the SNP calls and the genome annotation. We defined “speciation hotspots” as genes that satisfied the following two criteria: (1) can confidently (bootstrap > 95) separate *C. cecrops* and *C. isobeon* specimens into clades in phylogenetic trees by both the DNA sequences and the translated protein sequences encoded by them; (2) the divergence in protein sequences within at least one species is lower than 0.4%, which is close to the median divergence level over all the proteins. We identified the enriched GO terms associated with these “speciation hotspots” using binomial tests (m = the number of “speciation hotspots” that were associated with this GO term, N = number of “speciation hotspots”, p = the probability for this GO term to be associated with any gene). GO terms with P-values lower than 0.01 were considered enriched. Significantly enriched GO terms (p < 0.01) were visualized in REVIGO^81^. Important speciation hotspots were submitted to MESSA^82^ to perform secondary structure and disordered region prediction, domain identification and 3D structure prediction.

Similarly, we identified the “speciation hotspots” for *Pterourus glaucus* and *P. canadensis*. To compare the speciation hotspots for *Calycopis* and *Pterourus*, we attributed them to the orthologous groups identified by OrthoMCL (described above) and only the 6724 orthologous groups shared by both genera were analyzed. The significance level for the overlap in “speciation hotspots” between *Calycopis* and *Pterourus* was evaluated by a binomial test (m = number of orthologous groups that contain “speciation hotspots” from both genera, N = number of orthologous groups that contain “speciation hotspots” for a genus, p = probability for an orthologous group to contain “speciation hotspots” of another genus).

### Selection of nuclear DNA barcodes to identify *C. cecrops* and *C. isobeon*

We selected nuclear barcodes using the following procedure: First, we selected nuclear genes that could unambiguously classify the *C. cecrops* and *C. isobeon* specimens both by their sequences and the sequences of their protein products: we required the minimal interspecific divergence to be higher than the maximal intraspecific divergence by at least 0.1%. Second, the GO terms of these genes were analyzed and those that are associated with GO terms that are enriched in the speciation hotspots were considered as candidates for species identification. Third, the exons of these candidates were extracted and exons satisfying the following criteria were selected: (1) the N- and C-terminal 25 bp, which could be used to bind PCR primers were conserved with at most 2 variable positions across all specimens; (2) the difference between the minimal interspecific divergence (percent of different positions) and maximal intraspecific divergence was bigger than 1.0%; (3) the minimal number of different positions between species was bigger than the maximal number of different positions within a species by at least 2.

## Additional Information

**How to cite this article**: Cong, Q. *et al*. Complete genomes of Hairstreak butterflies, their speciation, and nucleo-mitochondrial incongruence. *Sci. Rep*. **6**, 24863; doi: 10.1038/srep24863 (2016).

## Supplementary Material

Supplementary Table S1

Supplementary Table S2

Supplementary Table S3

Supplementary Table S4

Supplementary Table S5

Supplementary Figures

## Figures and Tables

**Figure 1:**
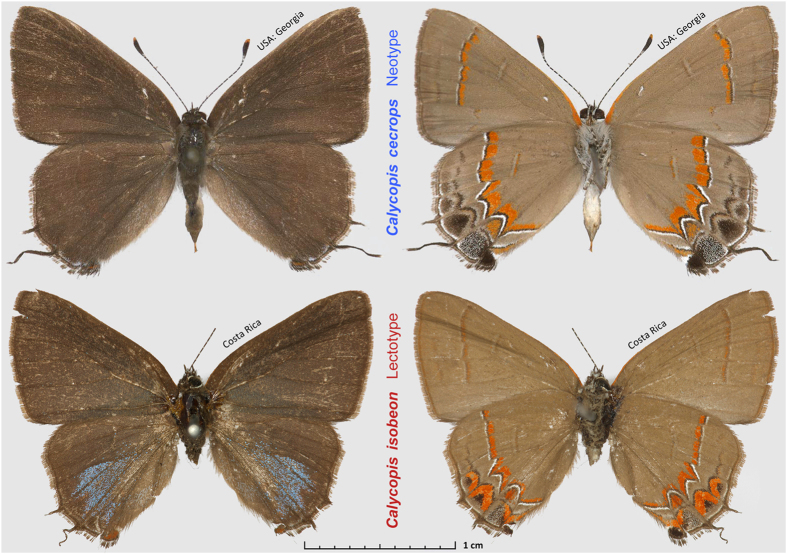
Primary type specimens of *Calycopis*species. Top: *C. cecrops* neotype, male, USA: Georgia, Chatham Co., Savannah, 30-May-1964, is in the National Museum of Natural History, Smithsonian Institution, Washington, DC, USA (USNM). Bottom: *C. isobeon* lectotype, male, Costa Rica, specimen # BMNH(E) 1669784 is in the Natural History Museum, London, UK (BMNH), images copyright of Trustees of the Natural History Museum, London, used with permission.

**Table 1:** Quality and composition of Lepidoptera genomes.

Feature	Pgl	Ppo	Pxu	Dpl	Hme	Mci	Lac	Bmo	Mse	Pxy	Cce	Pse
Size w/o gap (Mb)	361	218	238	242	270	361	290	432	400	387	689	347
Scaffold N50 (kb)	231	3672	6199	716	194	119	525	3999	664	734	233	257
GC content (%)	35.4	34.0	33.8	31.6	32.8	32.6	34.4	37.7	35.3	38.3	37.1	39.0
Repeat (%)	22.2	n.a.	n.a.	16.3	24.9	28.0	15.5	44.1	24.9	34.0	34.0	17.2
Exon (%)	5.11	7.79	8.59	8.41	6.19	4.34	7.24	4.07	5.34	6.47	3.11	6.20
Intron (%)	24.8	51.6	45.5	26.6	24.1	31.2	32.3	16.1	38.3	31.3	24.0	25.5
Genome size (Mb)	375	227	244	249	274	390	298	481	419	394	729	406
Heterozygosity (%)	2.3	n.a.	n.a.	0.55	n.a.	n.a.	1.5	n.a.	n.a.	~2	1.2	1.2
CEGMA (%)	99.6	99.3	99.6	99.6	98.2	98.9	99.6	99.6	99.8	98.7	100	99.3
CEGMA coverage by single scaffold (%)	86.9	85.8	88.8	87.4	86.5	79.2	86.8	86.8	86.4	84.1	85.3	87.4
Ribosomal Proteins (%)	98.9	98.9	97.8	98.9	94.6	94.6	98.9	98.9	98.9	93.5	98.9	98.9
*De novo* transcripts (%)	98	n.a.	n.a.	96	n.a.	97	98	98	n.a.	83	96	97
number of proteins (k)	15.7	12.3	13.1	15.1	12.8	16.7	17.4	14.3	15.6	18.1	16.5	16.5

*Pgl*: *Pterourus* glaucus; *Ppo*: *Papilio polytes*; *Pxu*: *Papilio xuthus*; *Dpl*: *Danaus plexippus*; *Hme*: *Heliconius melpomene*; *Mci*: *Melitaea cinxia*; *Lac*: *Lerema accius*; *Bmo*: *Bombyx mori*; *Mse: Manduca sexta*; *Pxy*: *Plutella xylostella*; *Cce*: *Calycopis cecrops*; *Pse*: *Phoebis sennae*.

Heterozygosity: Calculated as the percent of heterozygous positions detected by the Genome Analysis Toolkit (GATK) for *Pgl*, *Lac*, *Cce* and *Pse*; or taken from information in the literature for *Dpl*^6^; or estimated based on the histogram of K-mer frequencies for *Pxy*^16^,^47^.

n.a.: data not available.

**Table 2:** Quality of 8 *Calycopis* genomes.

Specimen (NVG-)	3306	3307	3515	3348	3431	3978	4166	3033
Coverage	78*	14.1	12.6	16.5	14.5	14.1	13.0	13.0
Mapped noncoding region (%)	100.0	88.7	88.2	84.4	84.2	83.1	83.2	82.5
Mapped coding region (%)	99.9	97.6	97.7	97.8	97.7	97.4	97.4	96.0
100% covered genes	15729	12950	12640	12278	12040	11504	11642	8113
90% covered genes	16446	15133	14958	14965	14876	14705	14717	13848
50% covered genes	16456	16109	16109	16130	16109	16089	16076	16062
Heterozygosity (%)	1.16	1.46	1.44	1.79	1.83	1.58	1.59	1.96
Heterozygosity (%) coding	0.56	0.64	0.65	0.82	0.84	0.71	0.71	1.04

*This is the coverage for paired-end libraries. The mate pair libraries for the reference assembly were prepared using DNA from both specimens 3306 and 3307.
