# Supplementary material for: Complete genomes of Hairstreak butterflies, their speciation, and nucleo-mitochondrial incongruence
Source: Sci Rep. 2016 Apr 28;6:24863. doi: 10.1038/srep24863 (PMC4848470; doi:10.1038/srep24863)
Supplement: Supplementary Figures [file srep24863-s6.pdf]

Complete genomes of Hairstreak butterflies, their speciation, and nucleo-mitochondrial incongruence

Qian Cong[2,*], Jinhui Shen[2,*], Dominika Borek[2], Robert K. Robbins[3], Zbyszek Otwinowski[2], and Nick V. Grishin[1,2,#]

[1]Howard Hughes Medical Institute, University of Texas Southwestern Medical Center, 5323 Harry Hines Boulevard, Dallas, Texas 75390-9050, USA.

[2]Department of Biophysics and Biochemistry, University of Texas Southwestern Medical Center, 5323 Harry Hines Boulevard, Dallas, Texas 75390-8816, USA.

[2]Department of Entomology, National Museum of Natural History, PO Box 37012, NHB Stop 105, Smithsonian Institution, Washington, D.C., 20013-7012 USA.

Email:

Qian Cong: qian.cong@utsouthwestern.edu

Jinhui Shen, Jinhui.Shen@UTSouthwestern.edu

Dominika Borek: dominika@work.swmed.edu

Robert K. Robbins: RobbinsR@SI.edu

Zbyszek Otwinowski: zbyszek@work.swmed.edu

Nick V. Grishin: grishin@chop.swmed.edu

* These authors contributed equally to this study.

#Correspondence: Nick V. Grishin

Supplemental Figures

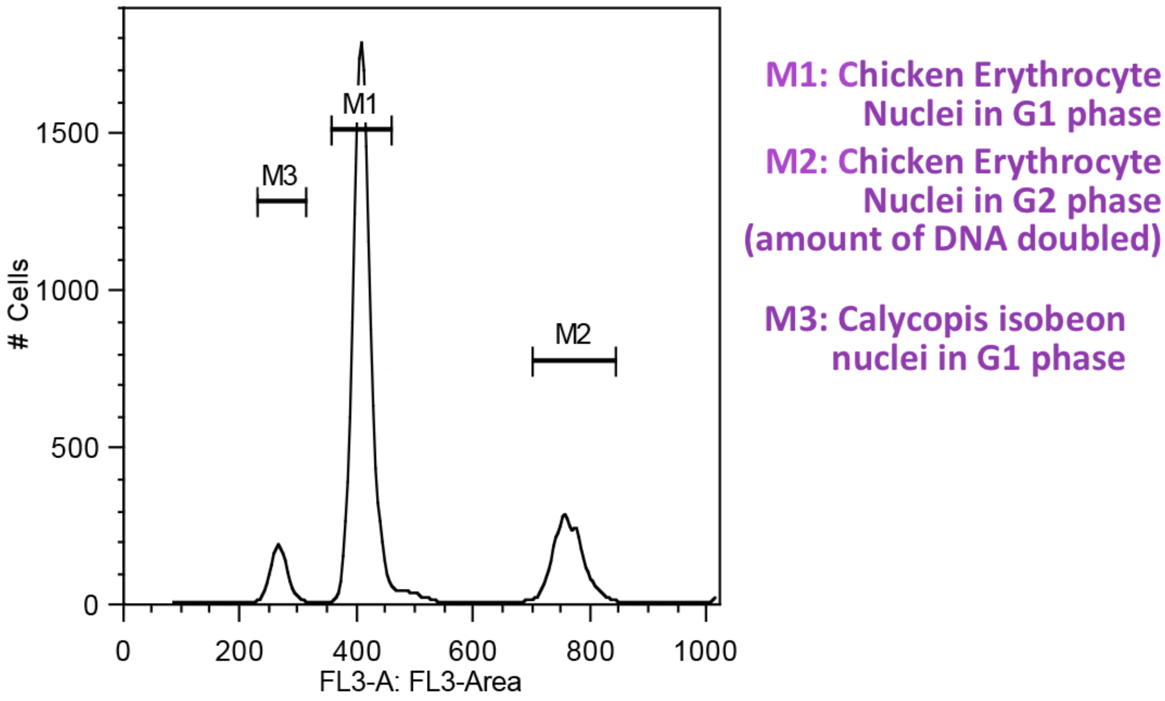

Figure S1. Estimation of genome size using flow cytometry.
The horizontal axis is the intensity of the fluorescence and it is linear correlated with the genome size. The Chicken Erythrocyte Nuclei was used as control. Based on this graph, the ratio in genome sizes of *Calycopis* versus Chicken is 260/410. The size of chicken genome is 1.2 Gbp, and therefore the estimated size of *Calycopis* genome is 760 Mbp.

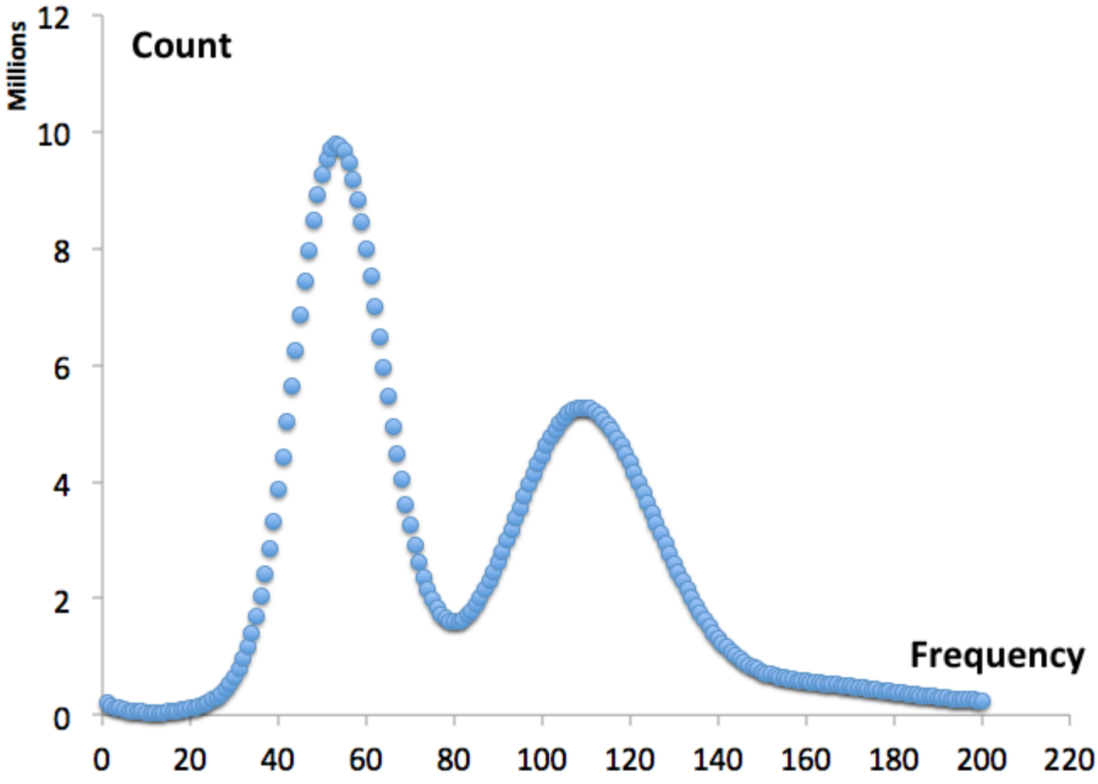

Figure S2. Estimation of genome size by histogram of 18-mer frequency in the reads. The reads after data processing have an average length of 137 bp and a total length of 81.5 Gbp. This graph shows that 18-mers from homozygous regions are covered approximately 109 times. Therefore, the estimated coverage for the genome is: coverage estimated from k-mer frequency x average read length / (average read length – k-mer length + 1) = 109 x 137/(137 - 18 + 1) = 124.4 fold[6], and the estimated genome size is 81,500,000,000/124.4 = 655 Mbp.

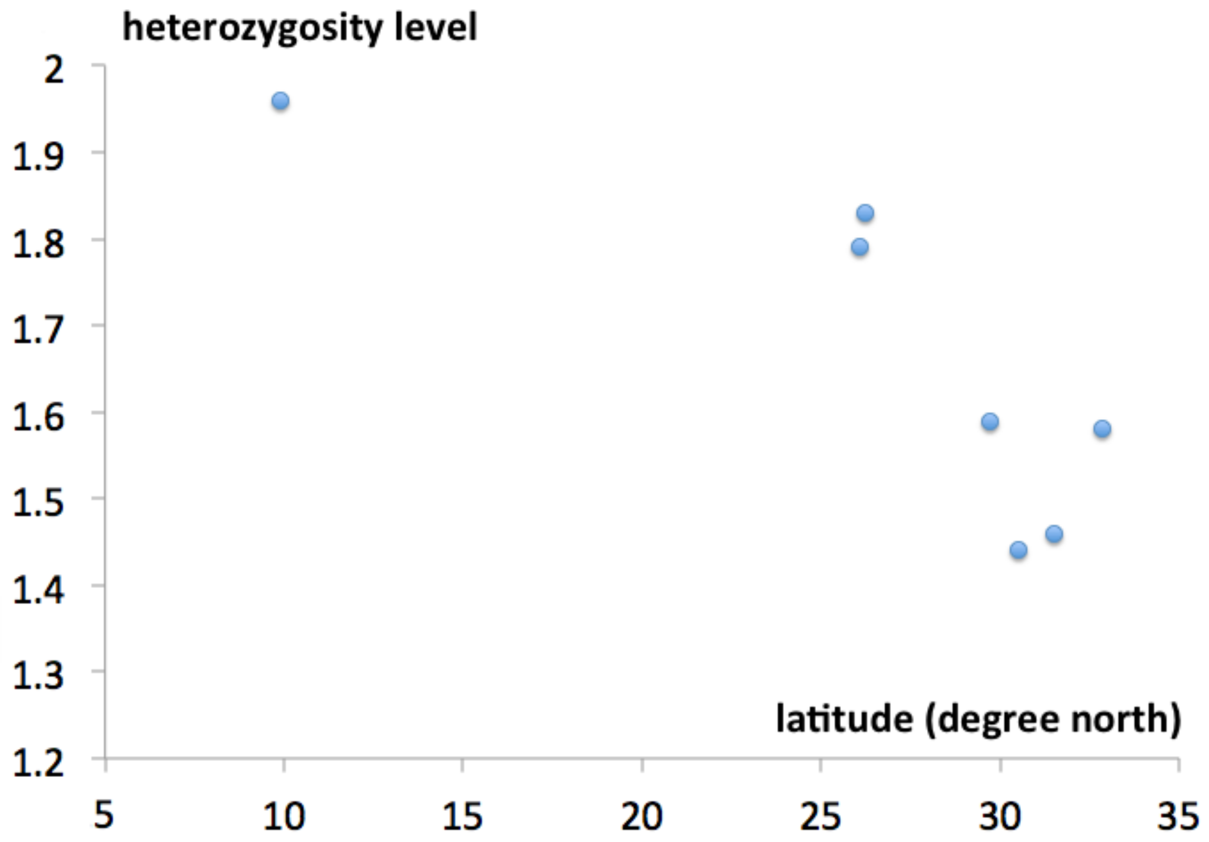

Figure S3. Correlation between the heterozygosity level and the latitude of the locality where the specimen is collected.

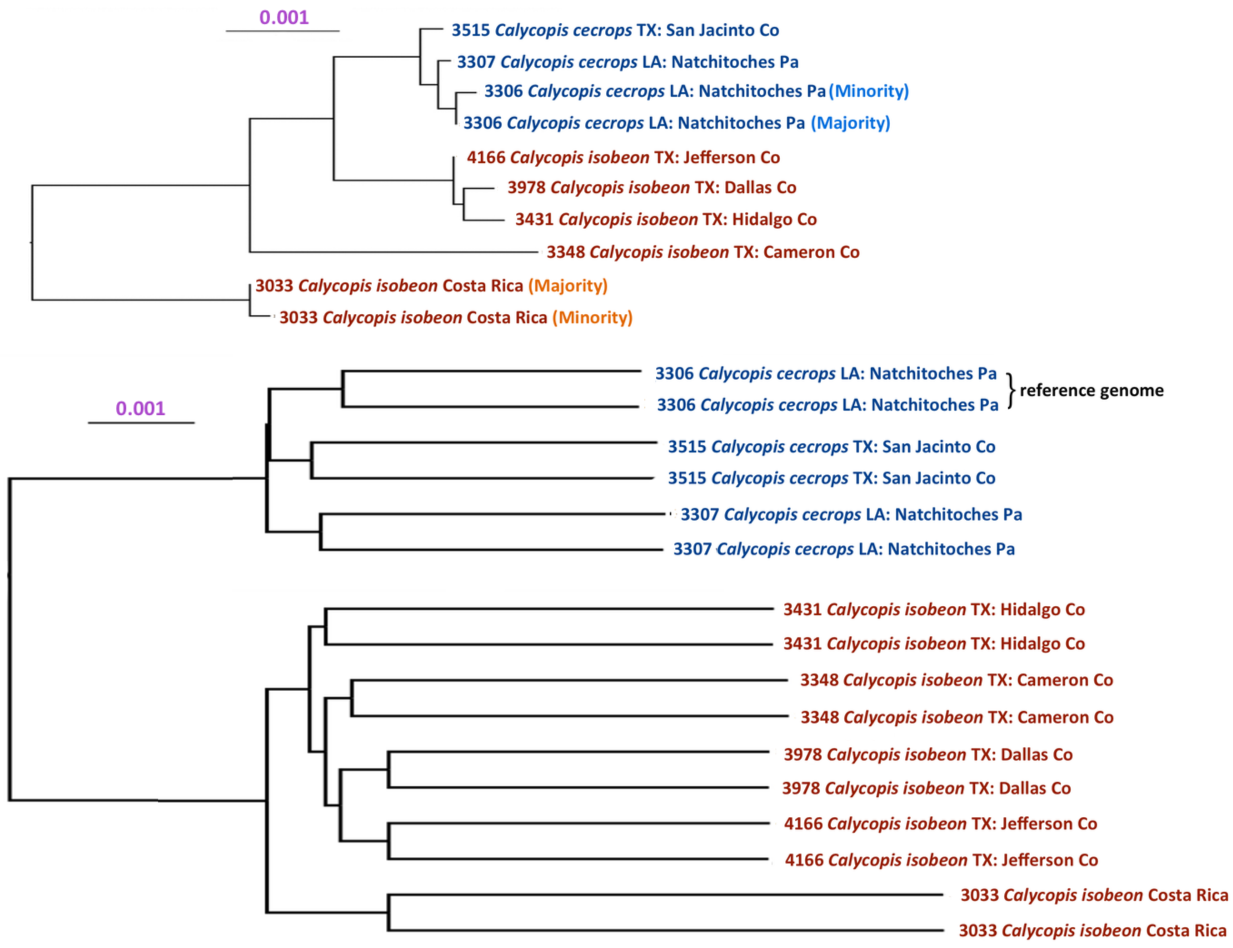

Figure S4. Incongruence between phylogeny inferred from mitochondrial and genomic DNA. Neighbor-joining trees based on the distance matrices calculated from the alignments of (a) mitochondrial genes (b) and nuclear genes. Specimen numbers, species names and localities are given. Two branches in nuclear trees corresponding to the same specimen refer to father and mother copy. Mitochondria of specimen 3033 and 3306 revealed two distinct types. Numbers by the nodes refer to bootstrap percentages.

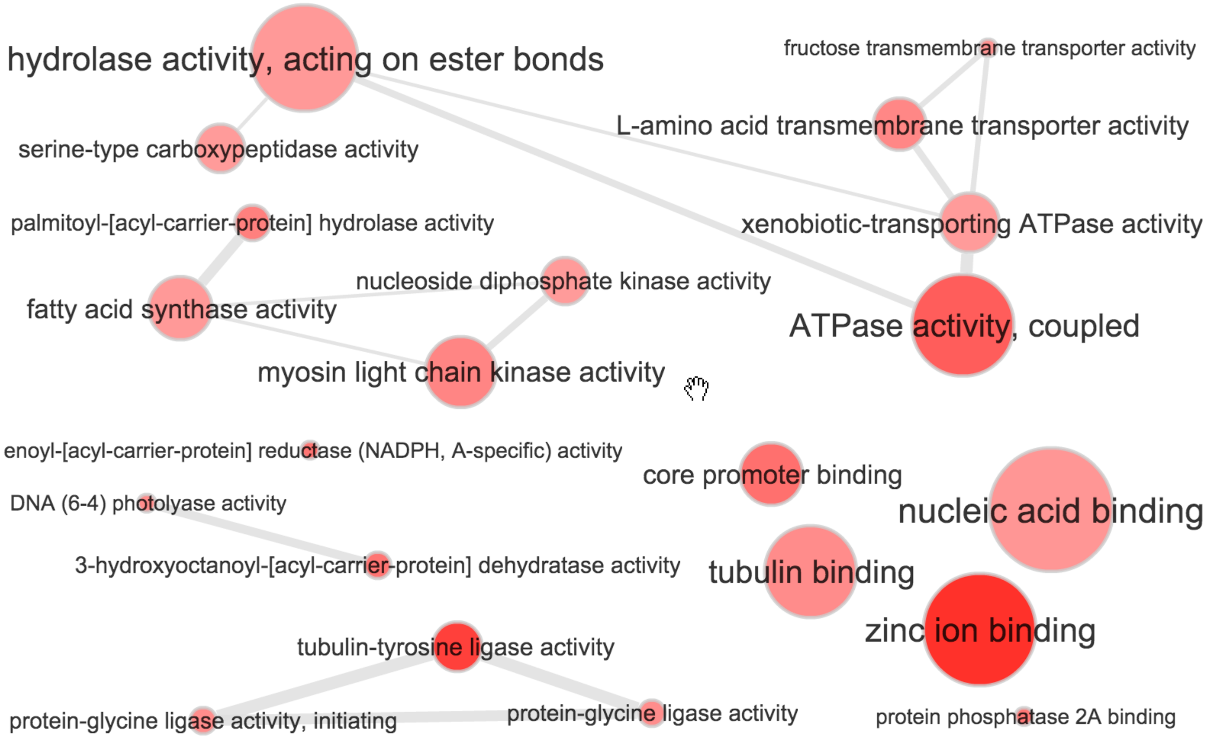

Figure S5. Significantly enriched GO terms that are associated with speciation hotspots of *Calycopis* in the category of molecular function. Each red dot represent one GO term as marked in the figure, and grey lines connect GO terms that are related and frequently associated with the same proteins. Darker color of the dots corresponds to higher level of significance and the size of the dots is positively correlated to the number of Drosophila proteins associated with this GO term.

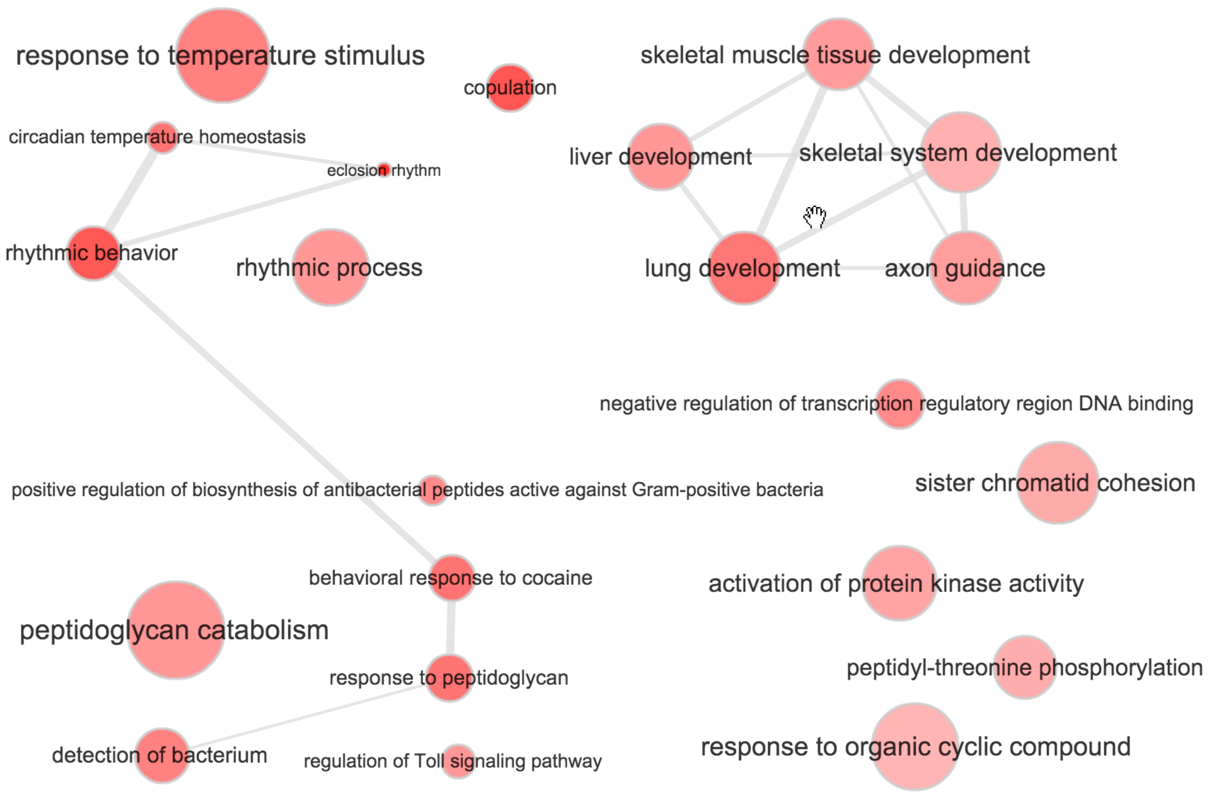

Figure S6. Significantly enriched GO terms associated with common speciation hotspots for *Calycopis* and *Papilio* species in the category of biological process. Each red dot represent one GO term as marked in the figure, and grey lines connect GO terms that are related and frequently associated with the same proteins. Darker color of the dots corresponds to higher level of significance and the size of the dots is positively correlated to the number of *Drosophila* proteins associated with this GO term.

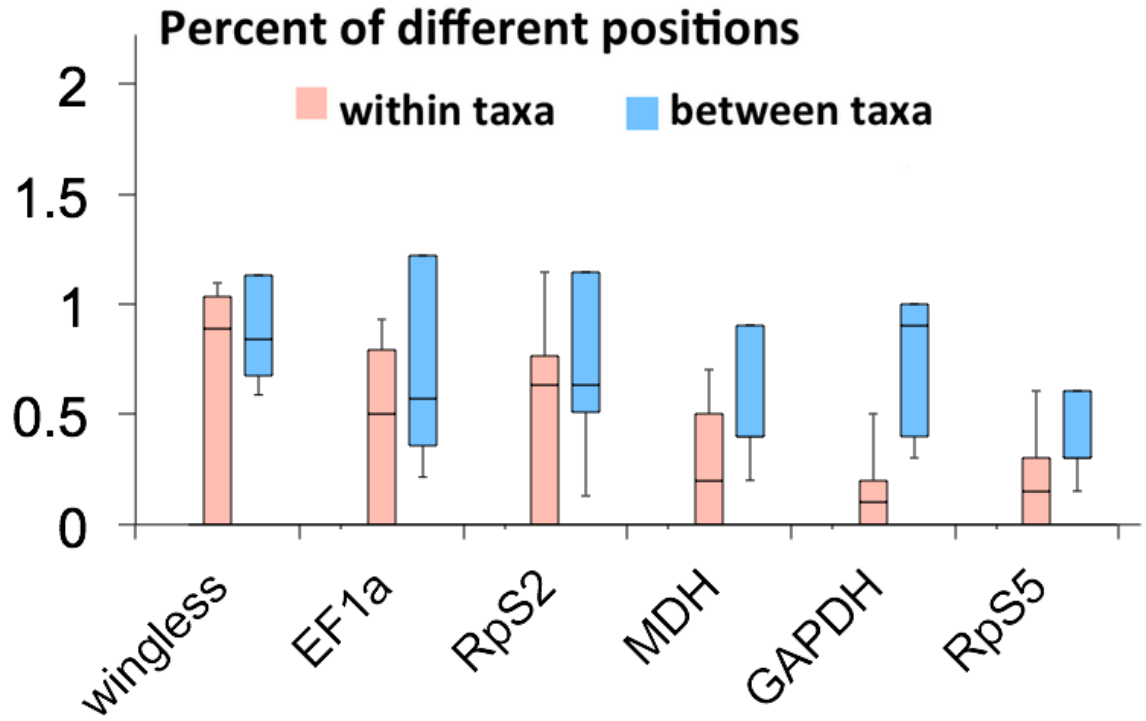

Figure S7. Divergence of previously selected nuclear markers for phylogeny within (red) and between (blue) *Pterourus* species (*Pterourus glaucus* and *Pterourus canadensis*). These markers are not suitable for distinguishing closely related species such as *Pterourus glaucus* and *Pterourus canadensis.*
